# The nexus between black and digital gold: evidence from US markets

**DOI:** 10.1007/s10479-021-04192-z

**Published:** 2021-07-22

**Authors:** Toan Luu Duc Huynh, Rizwan Ahmed, Muhammad Ali Nasir, Muhammad Shahbaz, Ngoc Quang Anh Huynh

**Affiliations:** 1 School of Banking, University of Economics Ho Chi Minh City, Ho Chi Minh City, Vietnam; 2 Chair of Behavioral Finance, WHU – Otto Beisheim School of Management, Vallendar, Germany; 3 IPAG Business School, Paris, France; 4 Department of Finance, University of Birmingham, Birmingham, UK; 5 Huddersfield Business School, University of Huddersfield, Huddersfield, UK; 6 School of Management and Economics, Beijing Institute of Technology, Beijing, China; 7 Institute of Business Research, University of Economics Ho Chi Minh City, Ho Chi Minh City, Viet Nam; 8 Department of Land Economy, University of Cambridge, Cambridge, UK

**Keywords:** Bitcoin, Copulas, Kendall plots, Partial cross-quantilogram, Oil market, US oil return, Tail risk and bootstrap test

## Abstract

In the context of the debate on cryptocurrencies as the ‘digital gold’, this study explores the nexus between the Bitcoin and US oil returns by employing a rich set of parametric and non-parametric approaches. We examine the dependence structure of the US oil market and Bitcoin through Clayton copulas, normal copulas, and Gumbel copulas. Copulas help us to test the volatility of these dependence structures through left-tailed, right-tailed or normal distributions. We collected daily data from 5 February 2014 to 24 January 2019 on Bitcoin prices and oil prices. The data on bitcoin prices were extracted from coinmarketcap.com. The US oil prices were collected from the Federal Reserve Economic Data source. Maximum pseudo-likelihood estimation was applied to the dataset and showed that the US oil returns and Bitcoin are highly vulnerable to tail risks. The multiplier bootstrap-based goodness-of-fit test as well as Kendal plots also suggest left-tail dependence, and this adds to the robustness of the results. The stationary bootstrap test for the partial cross-quantilogram indicates which quantile in the left tail has a statistically significant relationship between Bitcoin and US oil returns. The study has crucial implications in terms of portfolio diversification using cryptocurrencies and oil-based hedging instruments.

## Introduction

Uncertain price movements and risk contagions have been observed in the financial and energy markets due to unpredictability in economic development, discontinuity of economic policy and international geopolitical conflicts (e.g., see Li & Wei, 2018; Wei et al., 2017; Zhang & Wang, 2019; Mei et al., 2017; Fratzscher, 2012; and Wei et al., 2018). Investors typically select several hedging assets to offset their risk in the financial and energy markets. Crude oil and gold have long served as risk management tools to hedge against economic risks (Cunado et al., 2019; Lin et al., 2016; Ruan et al., 2016; Tang & Xiong, 2010; and Lei et al., 2019). In addition, cryptocurrencies have increasingly had a similar role since Bitcoin was introduced in 2008 (Nakamoto, 2008).

Bitcoin has been categorized as the ‘new gold’ or ‘digital gold’ by many financial media channels e.g., Bloomberg, CNN and Forbes. Furthermore, the Commodity futures trading commission (CFTC) has officially specified that virtual money is a form of commodity, in the same way as crude oil and gold. The market value of 1 Bitcoin as of 19 April 2021 was $56,617. Its value had sharply increased from $28,990 on 31 December 2020. Moreover, studies have confirmed that Bitcoin can be adopted for risk management and used as a short-term hedging tool in extreme market situations (Bouri et al., 2017; Demir et al., 2018; Eom et al., 2019). The digital currency innovation has changed the traditional concept of production function, which on three factors, i.e., land, labour and capital; however, the development of digital instruments and the proliferation of the internet have widened the landscape for financial modelling, economic risk and business. According to Schwab (2016), hedging through digital currencies is a revolutionary advance.

Our paper is linked to broad two strands of the literature. The first is research on financial modelling and risk management in relation to oil (the ‘black gold’), which has traditionally been used for hedging, safe haven and diversification (see studies on oil and similar asset classes in Sherman, 1986; Chua et al., 1990; Upper, 2000; Ciner, 2001; Hillier et al., 2006; Kaul & Sapp, 2006). The second strand is the literature on risk management, in particular for energy and environmental derivatives, through the use of new technologies like blockchain and cryptocurrencies; our study examines Bitcoin, which is widely considered a modern alternative safe-haven asset. Our paper provides fresh insight on the financial modelling and risk management of the key energy and environmental instrument, i.e. oil. Importantly, our analysis will give policymakers a better understanding and a new perspective on the roles of Bitcoin and oil as hedges and safe havens.

Our paper investigates the relationship between Bitcoin and US oil returns. Despite climate concerns, oil is one of the important sources of energy for household consumption and industries. Currently, oil is considered to be one of the main assets in the commodity markets for trading and hedging. Most economies in the world keep close track of oil price changes, because they can cause both microscopic as well as macroscopic problems, mainly through their effects on the equity market. High oil costs tend to lead to increases in the prices of other goods and services. Therefore, investors might be concerned about inflation. Oil prices also reflect the absorption costs for many manufacturing companies; hence, their profits and earnings might be affected. Consumer spending often declines in response to high oil prices, which leads to a decline in revenues for many companies as well. Thus, increases in oil prices often restrain further spending and investment. On 21 November 2018, the then US President, Donald J. Trump, posted on his Twitter account: “*Oil prices getting lower. Great! Like a big Tax Cut for America and the World. Enjoy! $54, was just $82. Thank you to Saudi Arabia, but let’s go lower!*”^1^ So, what happens if oil prices fall? Many industries may benefit from reduced production costs. Further, inflation will be stabilised. Hence, there is no pressure on interest rates, and more funds are available for investment. In this light, reductions in the oil price might encourage investment in attractive markets such as Bitcoin. Conversely, volatility in oil prices leads to uncertain policy; it also affects consumers’ and investors’ behaviours. Therefore, many studies have sought to relate oil prices to stock prices. Here, the work of Jones and Kaul (1996) is the foundation of theoretical and empirical studies of the nexus between oil and stock prices in the US and Canada; they argue that equity and oil markets have a statistical correlation. In the same year, Huang et al. (1996) found that oil futures contract returns significantly affect oil firms’ returns. Similarly, Faff and Brailsford (1999) and Sadorsky (1999, 2001) identify the relationship between oil and stock markets.^2^ However, few studies have investigated the co-movement of oil prices and Bitcoin. Therefore, this study attempts to contribute to the literature regarding the relationship between oil and Bitcoin.

Currently, cryptocurrencies are not only a trading market without transaction cost but also a decentralised system for initial coin offerings (ICO) and investment (e.g. futures contracts for Bitcoin^3^). In early 2021, the rapid increase in Bitcoin prices made many headlines in social media, leading to more interest from investors as well as warnings from authorities. Many earlier studies were inconclusive regarding whether Bitcoin is an investment asset or not (Briere et al., 2015). Therefore, we treat Bitcoin as a financial asset to test whether the oil price affects investment via inflation transmission. Bitcoin has become a ‘digital financial’ phenomenon, giving rise to much debate and many hypotheses have been proposed to explain movements in its value as well as the necessary economic methods. For example, Symitsi and Chalvatzis (2019) were the first to suggest the role of Bitcoin as a kind of commodity.

The US oil markets have also attracted much research attention, especially variations in demand and supply, because of their influence on the US economy (Sarwar et al., 2019). Lee and Ni (2002) emphasised that oil price shocks could have a variety of effects (positive or negative) on the economy, such as inflation, inflation expectations, governmental expenditures, and investor behaviours.^4^ Hamilton (2003) confirmed that oil shocks are associated with gross domestic product (GDP) growth. Moreover, Shapiro and Watson (1988) found that oil shocks could be used to predict exogenous political events in the Middle East. Therefore, we take into account US oil prices via their returns for model estimation.

Several previous studies have found bidirectional causality between two financial markets (Ajayi & Mougoue, 1996; Bae et al., 2003; Baele, 2005; Boyson et al., 2010; Jebran & Iqbal, 2016; Pan et al., 2007). However, they focused on the stock market, foreign exchange, or oil market. Few studies emphasise the spillover effect between the Bitcoin market and the oil market, both of which are volatile. Hence, a detailed investigation of the relationship between Bitcoin and the oil market is due, in part so that investors and policymakers can understand the co-movements of these assets.

Our results indicate that the Bitcoin and the US oil returns are highly exposed to tail-risk. The robustness of this result is enhanced by the additional use of the multiplier bootstrap-based goodness-of-fit test as well as Kendal plots, which also demonstrate the left-tail dependence. Furthermore, Selmi et al. (2018) explain the strong association between Bitcoin and oil returns in terms of Bitcoin’s similarities with gold, particularly from a risk taker’s point of view. Both are considered to be “counter-cyclical” to stocks or commodities and therefore “safe havens”. However, there are fundamental differences. First, Bitcoin is a relatively new market, and so investors have little experience of it; moreover, they also need advanced understanding of the associated data processing. Second, Bitcoin is much more volatile than gold. Third, the Bitcoin market has a thin volume, and is less organised and less regulated than the gold market. Fourth, the gold market is generally accepted to be a safe haven, by central banks, governments and individual investors, which is not the case for Bitcoin. Finally, the determinants of the gold price are well established, unlike those of Bitcoin, irrespective of its safe haven status. Therefore, one can suppose that investors who select gold as a safe haven are not the same as those who select Bitcoin.

This study makes a novel contribution in distinguishing risk management and hedging through financial modelling and fills a gap in the literature by relating the digital currency revolution to the energy markets. More specifically, the contribution is threefold: (i) this paper investigates the interdependence and spillover between the oil market and the Bitcoin trading market via their prices; (ii) this study employs different quantitative approaches—normal copulas, Clayton copulas and Gumbel copulas—to capture and confirm the dependence structure between Bitcoin returns and US oil returns; (iii) the robustness of the empirical findings is investigated by applying a stationary bootstrap for the partial cross-quantilogram. We find that Bitcoin and US oil returns have left-tail dependence and that Bitcoin can be used as a hedge against movements in oil prices. In addition, when investors select two kinds of assets in their portfolios, they should regularly inspect their co-movements.

The paper is organised as follows. Section 2 reviews the literature on the Bitcoin and oil market. Section 3 describes data collection. Section 4 discusses the quantitative techniques. Section 5 reports and discusses the empirical results. Section 6 presents the conclusions and policy implications.

## Literature review

Gold and crude oil are traditionally viewed as secure assets for investors to hedge market risks, and the recently introduced Bitcoin can be similarly viewed. The ability to obtain market risk information and to counter risk efficiently with these three major hedging assets, however, has not been investigated thoroughly with financial modelling and risk management techniques.

In our paper, we test the value of Bitcoin as a variable associated with the dynamic commodity variable of US oil returns. Most of the comparable studies on covariate research have focused on financial markets such as exchange rate, and stock, gold and oil prices. To date, very few studies have been conducted on the cryptocurrency markets. Huynh et al. (2018) indicated that there is a strong contagion risk among cryptocurrency markets. Their study, like ours, employed Kendall plots and the copulas approach but they did not indicate which quantile shows a strong correlation among these assets. Using GARCH and ECM, Van Wijk (2013) confirmed a long-run relationship between the Dow Jones index, the EUR/USD exchange rate and West Texas Intermediate (WTI) oil prices. Interestingly, all these variables significantly influenced Bitcoin returns, with a linear relationship between them. Nevertheless, more analysis is required to investigate tail-dependence and the use of quantitative techniques. More recent studies related to cryptocurrency and crude oil movements have highlighted cryptocurrency as a substitute medium of exchange due to its safety, transparency and cost-effectiveness (e.g. see Yuneline, 2019; Huynh et al., 2020; Jawadi et al., 2019; Ameur et al., 2020). Some of these examine the ARCH effects (e.g. Canh et al., 2019; Mensi et al., 2019) and have used an advanced method, namely time-varying vector-autoregression connectedness (TVP-VAR connectedness) (see for instance Dahir et al., 2019; Giudici and Abu-Hashish, 2019; Zeng et al., 2020).

In the present study, we investigate the relationship via the tail dependence structure. This methodological approach determines whether the two variables change in the same way or opposite directions in terms of the tail on their distributions. The left tail represents losses, the right tail gains. A normal distribution reflects random movements. Therefore, we use the dependence structure of the fat tail of the distributions of Bitcoin and US oil returns.

US oil is the subject of our research because it is an indicator of many economic signals. First, US oil is an important element of the cost of doing business in the United States and also a reference price for many countries. Moreover, higher inflation has a direct relationship with higher oil prices. When US oil prices increase, the impact will pass on to the consumer and businesses. Balke et al. (2002) asserted that rising US oil prices can cause financial stress and adversely affect monetary policies. However, a decline in US oil prices promotes investment. This can be theoretically explained by the study by Fisher (1896). Investors are likely to turn to investment assets other than oil when its price declines. Kang et al. (2014) examine how oil price shocks influence the US bond markets. Although their findings are interesting, they fail to explain why oil price shocks encourage financial leverage. Smyth and Narayan (2018) concluded that oil prices affect credit default swaps (CDSs), which are used to hedge by many companies. Therefore, oil prices determine the CDS spread, and so have an impact on financial markets.

Baur et al. (2018b) asserted that Bitcoin can also be used as a tool for hedging. Therefore, understanding the relationship between oil prices and Bitcoin is important before deciding on a hedging strategy. However, Bitcoin exchange markets are uncertain. All transactions are anonymous and are encoded. If oil prices decrease, what is the reaction of the Bitcoin exchange? Will there be a flight of capital to this new investment channel? Wan (2005) has contributed to the development of a theoretical framework as well as empirical evidence that decreases in the oil price increase stock prices via a dividend payout mechanism. However, the Bitcoin exchange markets have both demand and supply sides. The prices are driven by market signals. Based on the theory of interest as well as the earlier studies on capital flight, we investigate this relation in terms of dependence structure. Accordingly, below we review both Bitcoin studies and the literature on links between oil and Bitcoin prices.

Huhtinen (2014) examined the role of Bitcoin (both the demand and the supply sides) in the context of economic theory. Sapuric and Kokkinaki (2014) provided some empirical evidence that Bitcoin volatilities (i.e. its rate of exchange with other currencies) are considerably undervalued. Gronwald (2015) studied the sensitivity of the Bitcoin market to news in comparison with other markets. The author found that Bitcoin has extreme price movements and relies on demand-side factors while there was no evidence on the supply side. Briere et al. (2015) concluded that a portfolio with a small proportion of Bitcoin might be considered to be well diversified.

In relation to Bitcoin’s economic value, Cheah and Fry (2015) argued that it has zero fundamental value. Therefore, investments in this market are purely speculative. Another market efficiency perspective comes from a study by Jakub (2015), who showed that Bitcoin is likely to react quickly to publicly released information whereas the information related to macro-financial terms or economic context has a non-significant effect, and in support of this Baek and Elbeck (2015) found that the Bitcoin markets are driven more by the pressures of buyers and sellers than by economic fundamentals. Bouoiyour and Selmi (2015) argued that there is no evidence that Bitcoin used as safe haven for investors. Moreover, there is a largely speculative behaviour of Bitcoin, which shows the dependence structure with the Shanghai stock market and hash rate. Dyhrberg (2016a, 2016b) found that Bitcoin’s traits can be seen as a mixture of those of gold and the US dollar. As a type of investment, Bitcoin might be considered to have similar hedging abilities to gold. The implications of Dyhrberg’s work are that Bitcoin requires portfolio management, risk analysis as well as the evaluation of investor sentiment. In contrast, Ciaian et al. (2016a, 2016b) found that market forces drive Bitcoin prices and that macroscopic factors do not have a strong relationship with Bitcoin over the long term.

Moore and Stephen (2016) examined the case of the Central Bank of Barbados. The Bank held a small amount of Bitcoin, which significantly increased the return but had a non-significant influence on volatility. Moreover, Urquhart (2016) showed that the Bitcoin markets are presently inefficient but gradually moving towards being an efficient market. Jiang et al. (2018) reached a similar conclusion but Nadarajah and Chu (2017) argued that Bitcoin returns with simple power transformation do presently meet the efficient market hypothesis. Especially, Vidal-Tomás and Ibañez (2018) suggested that the Bitcoin market is semi-strong efficient but does not reflect monetary market news. When it comes to Bitcoin’s exchange rate against the US dollar and the euro, Sensoy (2019), using high-frequency data, showed that the latter markets are slightly more efficient.

Khuntia and Pattanayak (2018) presented evidence that Bitcoin has some efficiency/inefficiency characteristics that match the adaptive market hypothesis. A typical question regarding the economic characteristics of Bitcoin is ‘Does Bitcoin have a transaction cost?’ Koutmos (2018) in fact explained the relationship between Bitcoin returns and transaction activity. From another perspective, Blau (2018) could find no statistical evidence of speculative trading. Meanwhile, Takaishi (2018) investigated the skewness and multifractality of Bitcoin. Su et al. (2018) judged that Bitcoin can be used as a hedging instrument against market-specific risk. Feng et al. (2018) studied informed trading in the Bitcoin market.

Lintilhac and Tourin (2017) contributed an optimal dynamic pairs trading strategy and concluded that investors should be cautious because of limited liquidity and market depth. Balcilar et al. (2017) analysed the ability of Bitcoin volume to predict its returns and showed that the volume could forecast the return in the quantile range 0.25 to 0.75 whereas there was no evidence that volume affects volatility. Urquhart (2017) also confirmed that Bitcoin price clustering is significant, consistent with the hypothesis of Harris (1991). After testing a rich set of quantitative techniques, Katsiampa (2017) reported that AR-GARCH is the best model to explain Bitcoin volatility. In contrast, Ardia et al. (2019) found that MS-GARCH models outperformed AR-GARCH models in predicting Bitcoin volatility. Bariviera et al. (2017) showed that the Hurst exponent indicator changed in the early years of Bitcoin but was likely to be stable thereafter. Their study also implemented many quantitative methodologies.

The result from Baur et al. (2018a) contradicts the study by Dyhrberg (2016a). Bitcoin has different traits to both gold and the dollar. Therefore, its correlation characteristics are quite typical compared to the other assets. Additionally, Baur et al. (2018b) asserted that there is no relationship between Bitcoin and assets such as stocks, bonds, and commodities. Hence, those findings encourage the use of Bitcoin as a hedging instrument, even though many investors currently consider it to be purely a speculative investment or alternative currency.

Employing a series of tests, Aalborg et al. (2019) found that Bitcoin returns were not predicted by volatility, trading volume or number of Google searches for the term. Kristoufek (2013) also examined whether Bitcoin has a statistical relationship with online searches, and provides some insights on bubble behaviours as well as investors’ attention. Dastgir et al. (2019) employed the copula-based Granger causality test and indicated that it has tail dependence on Bitcoin and the number of Google searches. Nasir et al. (2019) employed parametric and non-parametric approaches to test the relationship between Bitcoin returns/volume and the Google search index. They confirmed that Google search engine use could forecast Bitcoin returns and volatility. An alternative approach by Panagiotidis et al. (2018) using LASSO regression confirms these findings. The Bitcoin returns can be predicted by search intensity (Google trend), gold returns and policy uncertainty. Furthermore, Yelowitz and Wilson (2015), in their analysis of Google search data, found that computer programming abilities and illegal activities are characteristics of those with an interest in Bitcoin, whereas professional investors do not consider Bitcoin to be an investment tool. Urquhart (2018) contributed to this literature by studying the attention given to Bitcoin; previous day volatility and volume of trading were both significant drivers of next-day attention. Corbet et al. (2018) reported no evidence of a persistent bubble in the Bitcoin market. Interestingly, Demir et al. (2018) provided new insight about economic policy uncertainty (EPU), which can well explain both Bitcoin returns and volatility (this index has a negative relationship with Bitcoin returns). Finally, Thies and Molnár (2018) employed a new quantitative technique called Bayesian change point integrated structural break to estimate Bitcoin returns.

In addition to the literature on Bitcoin itself, a series of studies have looked at the potential relationship between Bitcoin and oil prices. For instance, Van Wijk (2013) employed the vector error correction model to investigate the relationship between Bitcoin and different kinds of assets, including oil. The conclusion was that Bitcoin and oil prices are negatively related. Nevertheless, that study did not examine the tail risk on structural dependence. Wang et al. (2016) investigated whether oil prices and trading volume have an impact on Bitcoin prices. They found that oil prices have a negative effect on Bitcoin prices in the long run. Giudici and Abu-Hashish (2019) also examined the relationship between Bitcoin and different exchange markets. They investigated how Bitcoin and oil returns move and found that there is no significant partial correlation with Bitcoin prices. Their study only employs the network VAR process and it did not show the dependence structure and relationship on the tail structure, which could be interpreted as a spillover risk among these markets. Guesmi et al. (2019) suggested that Bitcoin might be exploited as a hedging strategy, because its addition could significantly minimise the risks of a portfolio with three kinds of assets (gold, oil and equities). Although this study indirectly mentioned the movement between Bitcoin and oil, it failed to explain how risks are transmitted between Bitcoin and oil, and vice versa. Noticeably, the study by Selmi et al. (2018) shed new light on the characteristics of Bitcoin, and whether it could be used to hedge for oil in comparison with gold. Their study indicated that the addition of Bitcoin can reduce portfolio risks with oil (as an element). Selmi et al. (2018) employed quantile-on-quantile regression as well as CoVaR to investigate their hypotheses. To examine the joint distribution of the assets, we use copulas as well as cross-quantilogram to capture the different distributions of Bitcoin and oil. This approach allows us to better understand the transmission mechanism through oil prices and other assets.

The above detailed review of the literature, with the key studies highlighted in Table 1, shows that few studies have examined the relationship between Bitcoin and oil prices. The present study fills this research gap by employing different types of quantitative techniques. The paper contributes to the current literature by reporting the dependence structure between Bitcoin and oil prices.

**Table 1:** List of recent studies of cryptocurrencies and crude oil

Year of publication	Names of authors, title of paper and journal	Empirical evidence
2019	Title of paper: Systematic risk in cryptocurrency market: Evidence from DCC-MGARCH model Authors: Nguyen Phuc Canh, UdomsakWongchoti, Su DinhThanh and Nguyen Trung Thong Name of Journal: Finance Research Letters	This study analyses the structural breaks and volatility spillovers of the seven largest cryptocurrencies: Bitcoin, Litecoin, Ripple, Stellar, Monero, Dash, and Bytecoin. The main results are as follows: Structural breaks exist in the cryptocurrencies The shifts spread from smaller cryptocurrencies (in market capitalisation) to larger ones Volatility spillovers indicate strong positive correlations among cryptocurrencies
2019	Title of Paper: Structural breaks and double long memory of cryptocurrency prices: A comparative analysis from Bitcoin and Ethereum Authors: Walid Mensi, Khamis Hamed Al-Yahyaee and Sang Hoon Kang Name of Journal: Finance Research Letters	Structural breaks affect the dual long memory of Bitcoin and Ethereum The authors applied four different ARFIMA-GARCH family models They found dual long memory present in Bitcoin and Ethereum returns and volatility Persistence decreases after considering long memory and switching states FIGARCH with structural breaks is the most appropriate technique for volatility predictions
2019	Title of Paper: Forecasting cryptocurrency returns and volume using search engines Authors: Muhammad Ali Nasir, Toan Luu Duc Huynh, Sang Phu Nguyen and Duy Duong Journal Name: Financial Innovation	The frequency of Google searches predicts positive returns and a surge in Bitcoin trading volume. Shocks to search values had a positive effect, which persisted for at least a week. The results have implications regarding the dynamics of cryptocurrencies/Bitcoins
2019	Title of Paper: Analysis of cryptocurrency's characteristics in four perspectives Author: Mirza Hedismarlina Yuneline Name of Journal: Journal of Asian Business and Economic Studies	This study examined the implications of cryptocurrency from the perspective of the nature of money, legal issues, the economy and Sharia. They found that cryptocurrency does not meet the criteria for a currency. Further, from the economic viewpoint, cryptocurrency does not fully match the characteristics of a currency due to high price volatility. From the Sharia perspective, cryptocurrency can be considered property (*mal*) but not as a monetary value (*thamanniyah*)
2019	Title of Paper: Do Jumps and Co-jumps Improve Volatility Forecasting of Oil and Currency Markets? Authors: Fredj Jawadi, Waël Louhichi, Hachmi Ben Ameur and Zied Ftiti Name of Journal: The Energy Journal	The authors found both markets show significant co-jumps driven by unanticipated macroeconomic news. Further, their model outperforms Corsi (2009)'s model in providing better forecasts. Specifically, co-jumps establish a key variable in forecasting oil price volatility
2020	Title of Paper: Diversification in the age of the 4th industrial revolution: The role of artificial intelligence, green bonds and cryptocurrencies Authors: Toan Luu Duc Huynh, Erik Hille and Muhammad Ali Nasir Name of Journal: Technological Forecasting and Social Change	The paper highlights the importance of AI and robotics stocks, green bonds, and Bitcoin in portfolio diversification Portfolios containing of these assets showed heavy tail dependence Volatility transmission is higher in the short term Bitcoin and gold are important assets for hedging and gold may act as a safe haven NASDAQ AI and general equity indexes are not good hedging instruments for each other

## Data

The daily data cover 5 February 2014 to 24 January 2019 for both Bitcoin prices and oil prices. The data on Bitcoin prices are extracted from coinmarketcap.com. US oil prices are collected from Federal Reserve Economic Data (https://fred.stlouisfed.org/). We use the logarithm of the return,^5^ following Miller (1972).^6^ The advantage of using the logarithm is that it has a more normal distribution. This is a prerequisite for the analysis of many multidimensional statistics as well as for deep learning techniques. Bitcoin and US oil prices are continuous-time stochastic processes, and so are continuous returns rather than discrete returns for each period. Thus, using the logarithm of returns (continuous returns) for these variables is better than using price or raw returns (discrete returns) (Hudson & Gregoriou, 2015).

Descriptive statistics are reported in Table 2. It can be seen that Bitcoin prices and oil prices are heavy-tail with high kurtosis values. The Bitcoin return has a positive mean but it is skewed to the left (negative skewness). Oil has a negative return with a -skew to the right. The interpretation of negative skewness (−0.37139) is that there are larger losses as the magnitude of the return increases. That is, overall, Bitcoin average returns over the research period are positive (0.000812) but they do also see large losses. Similar studies on Bitcoin have been conducted (e.g. Gronwald, 2015; Huhtinen, 2014; Sapuric & Kokkinaki, 2014). In contrast, the US oil means returns are negative (−0.00014) but have positive skewness (0.131320). This means that some of the US oil returns have a high positive value. The inverted characteristics of the movements of the two variables suggest there may be spillover, or contagion risks and gains from these investments. Because of their different distributions and characteristics, we employ copulas to capture the tail dependence between Bitcoin prices and oil prices, as they are able to capture dependence at any tail of the specific joint distribution (Xu & Brin, 2016). McNeil et al. (2015) also reported that copulas are better able to estimate results than simple maximisation methods because the copula function of flexibility evaluates the dimension of variables in different tails.

**Table 2:** Descriptive statistics

Variable	Mean	SD	Min	Max	Skewness	Kurtosis
Br (Bitcoin return)	0.000812	0.039296	−0.23757	0.225119	−0.37139	8.590618
Or (Oil return)	−0.00014	0.008458	−0.04832	0.049029	0.131320	7.944991

## Methodology

### The copulas^7^ approach

Many methods have been used to estimate the dependence structure between two variables, such as correlation coefficients, cointegration tests, Granger causality, dynamic conditional correlation, and generalised autoregressive conditional heteroscedasticity (GARCH) modelling and its variants (GARCH-MIDAS, FIGARCH, TGARCH, etc.). Some non-parametric quantitative techniques, such as Kendall (K)-plots, can be used to detect the existence of a dependence structure, and some parametric techniques, such as copulas, can capture how much one variable contributes to joint distribution on the tail (along with other variables). The main reason to use a non-parametric approach is to ensure the robustness of the results of a parametric technique (here, copulas). Nguyen and Bhatti (2012) and Huynh et al. (2018) examine parametric and non-parametric approaches.

A copula function generates the links from the *n*th-dimension of univariate marginal distributions to full multivariate distributions, which results in a joint distribution function of these random variables. Correlation is a scalar measure of dependence, and so cannot present the dependence structure of risks. On the other hand, copulas determine the dependence relationship and can indicate the position of tail dependence (left, right or normal).

Copulas are multivariate distribution functions. Assume that *H* is the distribution function of a *d*-dimensional random vector $X=\left({X}_{1},\dots ,{X}_{d}\right)$. Then

1 $$  H\left( x \right) = {\mathbb{P}}\left( {X \le x} \right) = {\mathbb{P}}\left( {X_{1}  \le x_{1} , \ldots ,X_{d}  \le x_{d} } \right) $$

in which, $$x = \left( {x_{1} , \ldots ,x_{d} } \right) \in {\mathbb{R}}^{d}$$.

The distribution function F_j_ of X_j_, $j\in \{1,\dots ,d\}$ can be derived from the multivariate distribution function H as $F_{j} \left( {x_{j} } \right) = H\left( {\infty , \ldots ,\infty ,x_{j} ,\infty , \ldots ,\infty } \right),{\text{~}}x_{j}  \in \mathbb{R}$. Therefore, we denote F_1_…F_d_ the univariate margins. Hofert (2018) introduced the simple approach of copulas, where the multivariate distribution function has standard uniform univariate margins, that is, U(0, 1) margins.

We use three kinds of copulas, namely normal, Clayton and Gumbel, to investigate the relationship between Bitcoin and US oil returns. Huynh et al. (2018) introduced the use of normal copulas, which do not have tail-dependence characteristics, through the formula:

2 $$  C_{\theta } \left( {u,v} \right) = \int\limits_{{ - \infty }}^{{\phi ^{{ - 1}} \left( u \right)}} {dx} \int\limits_{{ - \infty }}^{{\phi ^{{ - 1}} \left( v \right)}} {dy} \frac{1}{{2\pi \sqrt {1 - \theta ^{2} } }}\exp \left\{ { - \frac{{x^{2}  - 2\theta xy + y^{2} }}{{2\left( {1 - \theta ^{2} } \right)}}} \right\}  $$

where u and v are random variables and this parameter is in the range $(0\le \theta \le 1$). The Gumbel and Clayton copulas respectively represent right-tail and left-tail dependence. Jin (2018) summarised how to estimate the main characteristics of Gumbel and Clayton copulas (see Table 3).

**Table 3:** Clayton and Gumbel copulas: a summary. *Source*: Jin (2018)

Name	Copulas function	Parameter	Structure dependence
Clayton	$C_{C} \left( {u,v;\theta } \right) = C_{C} \left( {1 - u,1 - v;\theta } \right)$	$\theta$	Asymmetric tail dependence:$\lambda _{U} = 0,~\lambda _{L} = 2^{{ - 1/\theta }}$
Gumbel	$C_{G} \left( {u,v;\delta } \right) = exp\left( { - \left( {\left( { - \log u} \right)^{\delta } + \left( { - \log v} \right)^{\delta } } \right)^{{1/\delta }} } \right)$	$\delta$	Asymmetric tail dependence:$\lambda _{U} = 2 - 2^{{1/\delta }} ,~\lambda _{L} = 0$

Level of dependence in the left tail is denoted ${\lambda }_{L}$ and in the right tail ${\lambda }_{U}$, and u and v are random variables

Hofert (2018) demonstrated that the coefficient of lower and upper tail dependence of u and v can be expressed as:

3 $$ \lambda _{L}  = \lambda _{L} \left( {u,v} \right) = \mathop {{\text{lim}}}\limits_{{{\text{t}} \downarrow 0}} {\mathbb{P}}\left( {v \le F_{2} ^{ \leftarrow } \left( q \right){\text{|}}u \le F_{1} ^{ \leftarrow } \left( q \right)} \right), $$

4 $$   \lambda _{U}  = \lambda _{U} \left( {u,v} \right) = \mathop {{\text{lim}}}\limits_{{{\text{t}} \uparrow 1}} {\mathbb{P}}\left( {v > F_{2}^{ \leftarrow } \left( q \right){\text{|}}u > F_{1}^{ \leftarrow } \left( q \right)} \right),   $$

Let u and v be random variables with marginal distribution functions F_1_, F_2_. Then, $\lambda _{L} \left( {u,v} \right){\text{~}} \in {\text{ ~}}({\text{0}},{\text{1}}]$ (respectively $\lambda _{U} \left( {u,v} \right){\text{~}} \in ({\text{0}},{\text{1}}])$. This is the fundamental concept of estimating the parameters in these copulas.

The maximum pseudo-likelihood estimator is the most appropriate method for selecting copulas. If F_1_,…, F_d_ is known, the maximum likelihood estimator is also defined. However, it is likely to be unknown for univariate margins. Therefore, following Genest et al. (1995), Tsukahara (2005) and Kojadinovic and Yan (2010), the maximum pseudo-likelihood estimator is applied in our study:

5 $$  \theta _{{\text{n}}}  = \mathop {{\text{argsup}}}\limits_{{\theta  \in \Theta }} \sum\limits_{{{\text{i}} = 1}}^{{\text{n}}} {{\text{logc}}_{\theta } } \left( {{\text{U}}_{{{\text{i}},{\text{n}}}} } \right)  $$

U_i,n_ denotes univariate margins and n indicates the finite samples. For copula families, with the characteristics of finite samples and an asymptotic phenomenon, the maximum pseudo-likelihood estimator seems to be efficient. Therefore, our estimation for the selection of copula families is statistically based on the highest log-likelihood value. To ensure that our model has goodness-of-fit, we perform the multiplier bootstrap method for the selected copulas. Because we use it as our robustness test, we employ the five-step algorithm proposed by Genest and Rémillard (2008): (i) compute the pseudo-observations; (ii) estimate $\theta _{{\text{n}}}$ of ${\theta}$ from pseudo-observations; (iii) compute the test statistic ${S}_{n}^{gof}$; (iv) repeat steps (i) to (iii) for every $k\in \{1,\dots ,N\}$ if larger integer; (v) calculate the p-value for testing. Our methodology does not have any endogeneity issues. To deal with endogeneity issues (see e.g. Ullah et al., 2018, 2020).

### Bivariate copula Kendall plots

This concept draws from Genest and Boies (2003). The methodology used for continuous multivariate data meets the two basic requirements: (i) it is based on rank and (ii) maximally invariant statistics under transformations can be used without changing the copulas estimation results. The main use of bivariate copula Kendall plots is to rank the data, which are collected in a quantile–quantile-plot (QQ-plot) to test the normal features. A pair of variable ${(X}_{i},{Y}_{i})$ will transform into (${W}_{i}:n,H(i))$ within i = 1, 2…n. Therefore, the value of H_i_ is defined as follows:

6 $$ W_{i} :n = \mathop \smallint \limits_{0}^{1} \omega k_{0} \left( \omega  \right)\left\{ {K_{0} \left( \omega  \right)} \right\}^{{i - 1}} \left\{ {1 - K_{0} \left( \omega  \right)} \right\}^{{n - i}} d\omega $$

The ordered values of the bivariate distribution function are calculated by ${H}_{i}:={\widehat{F}}_{u,v}({u}_{1},{v}_{1})$. Thereafter, ${W}_{i}:n$ is the expected value of the order statistic from n random variables and ${{K}}_{0}\left({\omega }\right)={\omega }-{\omega }{l}{o}{g}({\omega })$, known as the corresponding density. Hence, a Kendal plot can be seen as the bivariate copula equivalent to the QQ-plot.

### Stationary bootstrap for the partial cross-quantilogram

The cross-quantilogram is a quantitative technique that obtains important econometric information for interpretation. It allows consideration of arbitrary lags and it does not meet moment conditions for a time series. Therefore, we further employ this approach to capture the properties of the joint distribution, especially tail dependence.

Linton and Whang (2007) first introduced the term ‘quantilogram’. The method predicts the correlogram pattern of two stationary time series in different quantiles. If it is extended to the analysis of many multivariates, this approach becomes the cross-quantilogram, as used by Han et al. (2016). The main use of the cross-quantilogram is to estimate parameters (denoted ${\rho }_{\tau }(k)$ with $\tau$ as a conditional or unconditional quantile) where there are two random variables $\left\{{x}_{t}\le \tau ({\tau }_{1})\right\}$ and $\left\{{y}_{t-k}\le \tau ({\tau }_{2})\right\}$ for an arbitrary pair of $\tau =({\tau }_{1},{\tau }_{2})$. The cross-quantilogram to estimate the dependence is defined as the cross-correlation of the quantile-hit process:

7 $$ \rho _{\tau } \left( k \right) = \frac{{E\left[ {\psi _{{\tau _{1} }} \left( {x_{t}  - \tau \left( {\tau _{1} } \right)} \right)\psi _{{\tau _{2} }} \left( {y_{t}  - \tau \left( {\tau _{2} } \right)} \right)} \right]}}{{\sqrt {E\left[ {\psi ^{2} _{{\tau _{1} }} \left( {x_{t}  - \tau \left( {\tau _{1} } \right)} \right)} \right]} \sqrt {E\left[ {\psi ^{2} _{{\tau _{2} }} \left( {y_{t}  - \tau \left( {\tau _{2} } \right)} \right)} \right]} }} $$

where

8 $$ \psi _{{\tau _{i} }} \left( {x_{t}  - \tau \left( {\tau _{i} } \right)} \right) = 1[y_{t}  < \tau \left( {\tau _{i} } \right)] $$

To measure dependence between two events $\left\{ {\tau _{{1,t}} \left( {\tau ^{l} _{1} } \right) \le x \le \tau _{{1,t}} \left( {\tau ^{h} _{1} } \right)} \right\}$ and $\left\{ {\tau _{{2,t}} \left( {\tau ^{l} _{2} } \right) \le y \le \tau _{{2,t}} \left( {\tau ^{h} _{2} } \right)} \right\}$ for arbitrary quantile ranges $[\tau ^{l} _{1} ,\tau ^{h} _{1} ]$ and $[\tau ^{l} _{2} ,\tau ^{h} _{2} ]$, an alternative calculation of the cross-quantilogram is defined:

9 $$ \psi _{{[\tau ^{l} _{i} ,\tau ^{h} _{i} ]{\text{~}}}} \left( {x_{t}  - \tau \left( {[\tau ^{l} _{i} ,\tau ^{h} _{i} ]{\text{~}}} \right)} \right) = 1\left[ {[\tau ^{l} _{i}  < y_{t}  < \tau ^{h} _{i} } \right] - (\tau ^{l} _{i}  - \tau ^{h} _{i} ) $$

Here, we apply the method used by Politis and Romano (1994a, 1994b) to estimate the critical values by the stationary bootstrap. The stationary bootstrap sampling process treats stationary conditions only. The cross-quantilogram based on the stationary bootstrap is defined as:

10 $$ \rho _{\tau } \left( k \right) = \frac{{\mathop \sum \nolimits_{{t = k + 1}}^{T} \left[ {\psi _{{\tau _{1} }} \left( {x^{*} _{t}  - \tau _{t} ^{*} \left( {\tau _{1} } \right)} \right)\psi _{{\tau _{2} }} \left( {y^{*} _{{t - k}}  - \tau _{{t - k}} ^{*} \left( {\tau _{2} } \right)} \right)} \right]}}{{\sqrt {\mathop \sum \nolimits_{{t = k + 1}}^{T} \psi ^{2} _{{\tau _{1} }} \left( {x^{*} _{t}  - \tau _{t} ^{*} \left( {\tau _{1} } \right)} \right)} \sqrt {\mathop \sum \nolimits_{{t = k + 1}}^{T} \psi ^{2} _{{\tau _{2} }} \left( {y^{*} _{t}  - \tau _{t} ^{*} \left( {\tau _{2} } \right)} \right)} }} $$

## Empirical findings

The first step is to test the stationary properties of Bitcoin and US oil returns. For this purpose, we apply ADF (Dickey & Fuller, 1981) and PP (Phillips & Perron, 1988) unit-root tests. The empirical results of ADF and PP unit root tests are reported in Table 4. We find that Bitcoin and oil prices in the USA are level with intercept and indicates 1% level of significance. This shows that Bitcoin and the US oil returns are integrated at I(0), which leads us to further empirical analysis.

**Table 4:** ADF and PP unit toot tests

Variables	Augmented Dickey-Fuller	Phillips-Perron
br (Bitcoin return)	−42.409***	−42.412***
or (Oil return)	−45.283***	−45.297***

*, **, and *** denote the significance at the 10%, 5%, and 1% levels, respectively

Concomitantly, we also performed the ARCH–LM, which is is Engel's LM test, for heteroskedasticity, conducted using 10 lags. The results show that, at the 1% significance level, all ten tests reject the null hypothesis that the errors are not autoregressive conditional heteroskedastic. Therefore, we found the GARCH effects for our variables.^8^

After confirming that Bitcoin and oil prices in the USA are integrated at I(0), we estimate the copula parameters to fit the value based on the maximum pseudo-likelihood of our 1815 observations for two dimensions. With the method of optimisation converged, Table 5 presents the copula parameters and maximised log-likelihoods. We chose the most appropriate copulas based on the highest log-likelihood values. Notably, Clayton copulas were usually the most appropriate in terms of the log-likelihood criterion (see Genest et al., 1995; Tsukahara, 2005; Kojadinovic & Yan, 2010). This also shows that the left-tail dependence has specific characteristics for the pair of Bitcoin and the US oil returns. It indicates the probability of the co-occurrence of extreme downside events for the two kinds of investments. That is, when Bitcoin suffers a loss in its return, the US oil market is also likely to experience a negative return. Interestingly, in recent years, the US government has tended to expect oil prices to decrease. Currently, US oil prices are experiencing a sharp pullback, which leads to negative returns as well. Therefore, investors are likely to change their capital investment flows from cryptocurrencies to another type of asset (e.g. Bitcoin as an alternative investment). Unexpectedly, this phenomenon leads to a decline in Bitcoin prices—a ‘crash’ after a ‘hot bubble’. In brief, we can see that the capital flow from the oil market to the Bitcoin market is ineffective. It is likely to have an extreme downside in returns when these kinds of assets experience a negative shock.

**Table 5:** The estimated parameters for paired bitcoin and US oil returns

Copulas	$\theta$	Log-likelihood
Normal	0.001	0.001
Clayton	0.01	**0.141**
Gumbel	1	−8.734e−07

The bold numbers represent the chosen Copula for further analyses

Assume that $\complement$ is a Normal, Clayton or Gumbel Copulas. Afterwards, the algorithm fits two trivariate copula families to our data for generating the parameters and log−likelihood values

We used the sorted-out approach to analyse data from a period of several days in which there were negative co-movements of Bitcoin and oil returns. There were 64, 37, 59, 67 and 64 days in the years 2018, 2017, 2016, 2015 and 2014, respectively, when both Bitcoin and oil returns had negative returns. After reaching a peak of $19,783.06 per one Bitcoin, this alternative investment crashed in 2018. This was related to the collapse of one of the biggest Bitcoin exchanges (Bitconnect) in 2018. The year 2018 also saw trade tensions between the US and China, as well as political disruptions in Europe. These factors suggested the prospect of a global recession, which led to a decline in US oil prices. Hence, the capital flows of investors between the Bitcoin and oil markets were inefficient. Based on our findings, we suggest the downside co-movements of these assets was due to the occurrence of bad events that were highly likely to lead to other bad events. Our results contribute to the empirical evidence that Bitcoin and US oil markets share a downside trend and experience a shock in negative returns (the related studies on Bitcoin and commodities include Dyhrberg, 2016a, 2016b; Ciaian et al., 2016a, 2016b; and Urquhart, 2016).

Following the suggestions made by Genest et al. (2009) and Fermanian (2013) in their literature reviews, we test whether our copulas belong to at least one family of copulas or not. The null hypothesis is $H_{0} :{\complement } \in \mathbb{C}$ versus $H_{A} :{\text{~}}{\complement }{\text{~}} \notin {\text{~}}\mathbb{C}$. We use the p-value methodology from Genest and Rémillard (2008) to assess the multiplier bootstrap value. Our null hypothesis is constructed as $H_{0} :{\text{~}}{\complement }{\text{~}} \in {\text{~}}\mathbb{C}_{{Clayton}}$ versus $H_{0} :{\text{~}}{\complement }{\text{~}} \notin \mathbb{C}_{{Clayton}}$.

Based on the p-value, we fail to reject the null hypothesis of bivariate financial log-returns between Bitcoin and US oil prices. Therefore, our estimation is Clayton copulas (Table 6). This confirms the above finding that Bitcoin and US oil returns have co-movements on the downside. Overall, our results indicate that Bitcoin returns parallel US oil prices due to contagion. Thus, there is a link between the two markets. Investors are likely to invest in one of them at the same investigation. Therefore, when a negative extreme event happens, both markets are likely to see a ‘flight to safety’, as mentioned by Bernanke et al. (1994). Furthermore, we suggest that negative information in relation to US oil might lead to a crash in the Bitcoin market. This reflects information efficiency, given that the same information transmits to both markets. The US oil markets heavily rely on the United States economy, which also causes uncertainty in the Bitcoin exchange markets.

**Table 6:** Multiplier bootstrap−based goodness−of−fit test of the Clayton Copula

Test	Parameter	Statistics	P−value
Goodness−of−fit	0.012	0.012	0.930

*, **, and *** denote the significance at the 10%, 5%, and 1% levels, respectively

### Bivariate copula Kendall plots

**Fig. 1:**
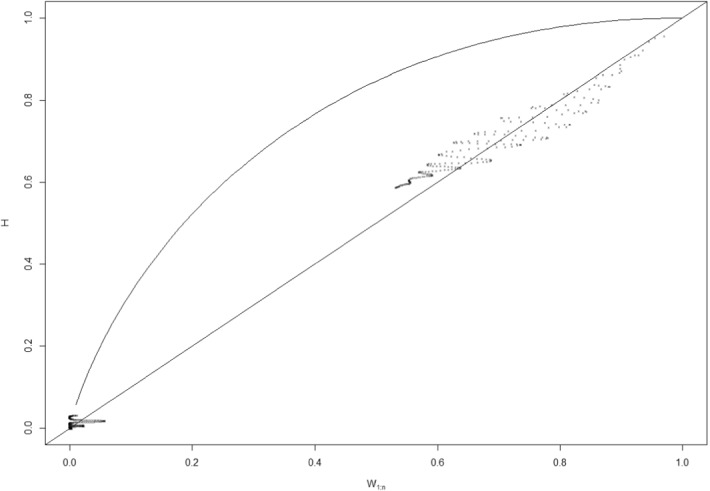
Bitcoin and US oil returns on bivariate copula Kendall plots. *Source*: R implemented

We employ a rich set of quantitative techniques to determine whether Bitcoin and the US oil returns share their dependence structure or not. The bivariate copula Kendall plots show that this pair of assets have a common dependence structure. Genest and Boies (2003) conclude that variables’ deviations from the 45° degree diagonal line can show dependence. Points of the Kendall plots above the diagonal line reveal positive dependence, and vice versa for negative dependence. Based on the results presented in Fig. 1, we can conclude that the two variables have a common dependence structure. Moreover, there are more deviations below the diagonal line, which demonstrates that Bitcoin and US oil returns have a negative dependence, according to the non-parametric approach. Our results, once again, confirm that there is a contagion risk from the US oil and Bitcoin exchange markets. We find that the contagion risk attracts the attention of investors who want to move from a downside asset to another asset (capital flight). However, this can cause volatility spillovers for the alternative investment.

This approach also acts as a robustness check on the prior findings, as it again shows that Bitcoin and US oil returns have a dependence structure.

### Stationary bootstrap for the partial cross-quantilogram

We examine the stationary bootstrap for partial cross-quantilogram for two variables (Bitcoin and US oil returns). Using the stationary bootstrap in Politis and Romano (1994a, 1994b), our results are statistically simulated by the number of repetitions for the stationary bootstrap, up to 10 times. Due to the computational burden, we compute the stationary bootstrap for the partial cross-quantilogram as left-tailed quantile levels $\tau _{i}  = 0.05,0.1,0.2,{\text{~}}0.3,{\text{~}}0.4{\text{~}}and{\text{~}}0.5$ and for the two lagged values (the first order and the second-order).

In our bootstrapping process, we set the number of repetitions for stationarity at 10 with each trial of 10,000 bootstrap replications. The rules of Politis and White (2004) and Patton et al. (2009)were followed to decide these tuning parameters. From Table 7, it can be seen that the stationary bootstrap for the cross-quantilogram is significant in the quantile ${{\tau }}_{{i}}=0.1$ for the parameter, which meets the 5% level of significance. There are non-significant parameters in the second-ordered lag value (the spillover effects). There is evidence that the effects of left-tail dependence in the stationary bootstrap for cross-quantilogram are unsteady. Because this is left-tail dependence, we do not consider the sign of the parameters, and so it is apparent only that there is a risk spillover (in one direction or other) between Bitcoin and US oil returns. Interestingly, there is a decrease in the magnitude of the cross-quantilogram by each quantile: the higher the quantile, the lower the cross-quantilogram parameter. Thus, a shock is likely to have a significant impact on both Bitcoin and US oil returns in the quantile ${{\tau }}_{{i}}=0.1$. Using this quantitative technique, we also examine the quantile for the left-tailed dependence between our two variables. This result confirms the previous findings and constitutes further evidence in a specific quantile for contagion risk among these assets.

**Table 7:** The stationary bootstrap for cross−quantilogram analysis

${{\tau }}_{{i}}$	Critical value	${\widehat{\rho }}_{\tau }\left(k\right)$ Cross−Quantilogram
*Lag value (1)*		
0.05	[−0.109; 0.041]	0.102
0.1	[−0.080; 0.078]	**0.070**
0.2	[−0.063; 0.000]	0.046
0.3	[−0.057; 0.020]	0.035
0.4	[−0.055; 0.022]	0.028
0.5	[−0.043; 0.020]	0.023
*Lag value (2)*		
0.05	[0.000; −0.005]	−0.005
0.1	[−0.005; 0.000]	−0.007
0.2	[−0.007; $\cong 0$]	−0.011
0.3	[−0.010; 0.000]	−0.015
0.4	[−0.012; 0.000]	−0.019
0.5	[−0.017; $\cong 0$]	−0.023

The bold numbers represent the chosen Copula for further analyses

We use a significance level of 5% as the criterion for a critical value. The parameters ${\widehat{\rho }}_{\tau }(k)$ are estimated to detect the predictability of the Bitcoin and the US oil returns in the left−tailed

Our findings are also in line with those of Wang et al. (2016). However, Wang et al. (2016) found a negative relationship whereas we estimate co-movements in the left-tail distribution. This means that the Bitcoin returns and the US oil returns are likely to see an extreme loss at the same time, which is considered to be contagion risk or spillover risk (Nguyen & Bhatti, 2012). Our findings are similar to those of Cappiello et al. (2006), who found a spillover (with negative effect) from the US oil market, although their study looked at stocks and bonds rather than a cryptocurrency.

However, our findings are in contrast to those reported by Selmi et al. (2018), who suggested that Bitcoin should be a good hedging instrument. The main reason for the conflicting findings may be that our study covers the period up to 24 January 2019, which was before the crash. Furthermore, their study emphasised the relationship of Bitcoin to oil and gold. Therefore, in our study, we suggest empirical evidence with different quantitative techniques that Bitcoin and oil returns have a left-tail dependence structure, which might trigger spillover or contagion risks in the downside trend.

### Robustness check

In this section, we use an advanced method, namely time-varying vector-autoregression connectedness (TVP-VAR connectedness), without taking into account the tail dependence to see how these commodities are connected to each other. The detailed methodology is set out in Huynh et al. (2020) and Pham and Huynh (2020).

**Fig. 2:**
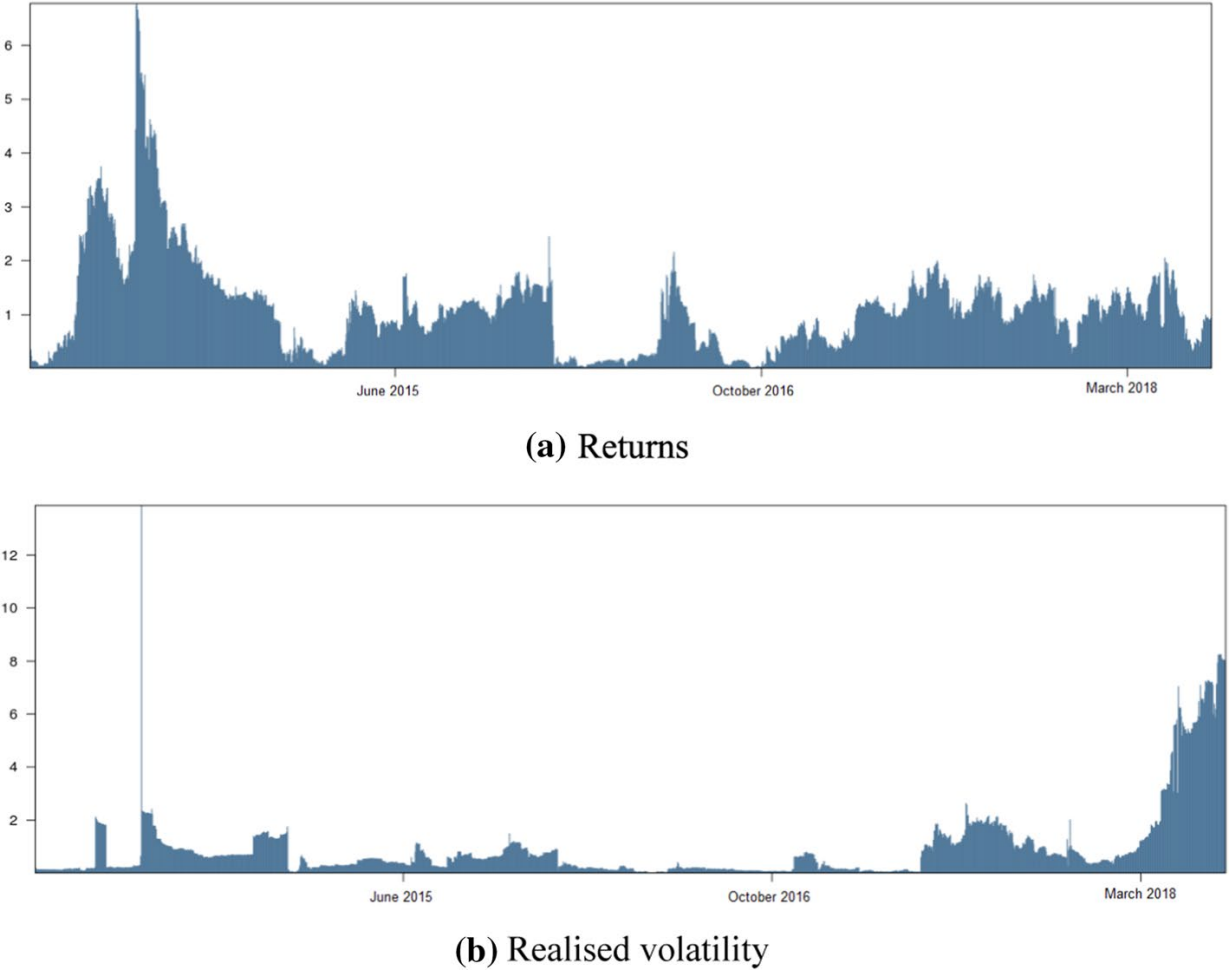
The total connectedness in return and volatility between Bitcoin and oil returns. **a** Returns, **b** Realised volatility. *Notes*: The total return and volatility connectedness values are 0.9% and 1.3%, respectively

In line with the previous findings and results, these commodities exhibit higher connectedness in their volatility, which supports the left-tail dependence of the losses on both of them. Overall, our findings shed new light on the relationship between oil and Bitcoin in terms of returns and volatility. Accordingly, the return connectedness exhibits a high persistent trend over the period 2015 to 2018 (Fig. 2). Bitcoin and crude oil saw the highest return spillover effect at the beginning of 2013. This marked a boom in Bitcoin when traded at the exchange. However, the volatility spillover effect reached a high in 2017, when Bitcoin saw a market crash.

We then expanded our dataset through to April 2021 to cover the pandemic, to examine the effect of that public health crisis on the connectedness of the two assets. In addition, we also employed the new measure to adjust the total connectedness of the two assets.

**Fig. 3:**
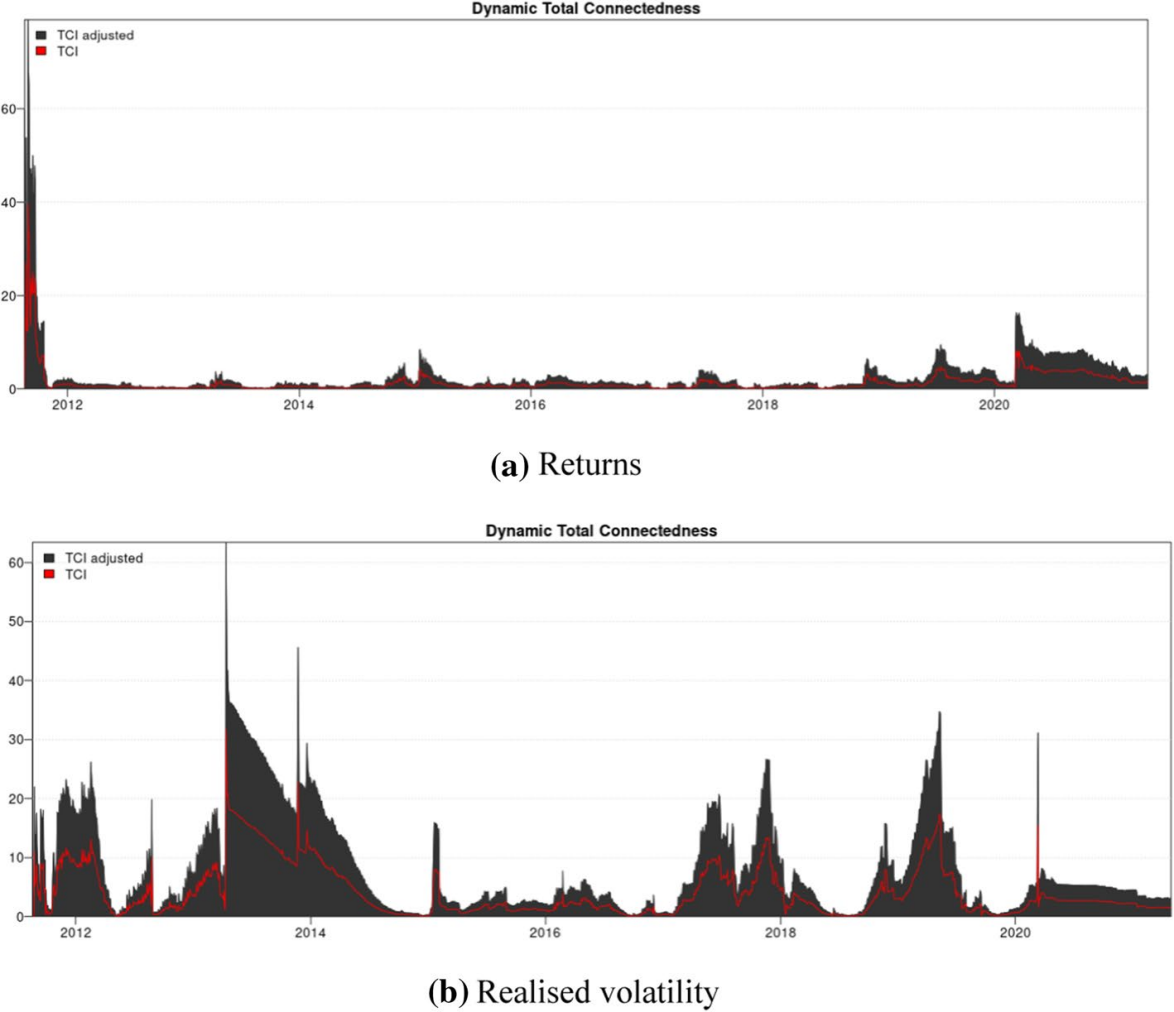
The total connectedness in return and volatility between Bitcoin and oil returns with the extended data. **a** Returns. **b** Realised volatility. *Notes*: We adjusted the total connectedness index by using the approaches by Gabauer (2021) and Chatziantoniou and Gabauer (2021)

Figure 3 plots the spillover effects between the two assets in terms of return and volatility over the extended period, that is, from 2012 to 2021. We find that the volatility linkage is more pronounced in three periods: the cryptocurrency boom (2013), the market crash (2017–2018), and the pandemic period (2020).

**Fig. 4:**
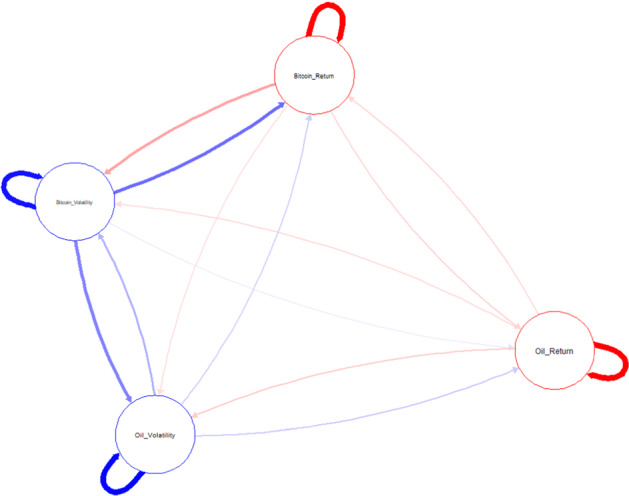
Diagram to illustrate the connectedness between Bitcoin and oil. *Notes*: Thicker lines indicate a higher degree of shock. Red and blue respectively represent the ‘sending’ and ‘receiving’ shocks among variables.

Figure 4 illustrates how the returns and volatility exhibit the spillover in the network. We can see the directions of shocks: sending or receiving from ‘starting’ points.

Finally, we examine the effects of the COVID-19 pandemic on the total return and volatility connectedness, as presented in Table 8.

**Table 8:** Effect of the COVID−19 pandemic on connectedness

Variables	Return connectedness	Volatility connectedness
Log(COVID−19 cases)	0.211*** [2.99]	
Log(COVID−19 deaths)		0.129*** [2.56]
Constant	3.806*** [4.22]	4.652*** [6.55]
F−stat	8.92***	6.54**
R−squared (%)	2.96	2.31

* < 0.1, ** < 0.05, *** < 0.01

The robust SE are in brackets

It is important to note that the severe shock of the pandemic plays an important role in the connectedness of Bitcoin and oil. The higher the number of cases, the higher is the degree of spillover of these two assets in terms of returns. This implies that public health shocks can predict a higher level of connectedness between two assets. Therefore, the portfolio diversification strategy should account for the fact that the systemic risk of two asset classes also increases. Our study is also consistent with earlier studies that have found that the COVID-19 pandemic is associated with contagious risk (Huynh, Foglia, Doukas, 2021; Liu et al., 2021).

## Conclusion and implications

This study investigates US oil returns and links them with a financial hedging instrument, i.e., Bitcoin. A risk management strategy identifies possible risks, problems or uncertainties before they occur. For example, stakeholders can design a framework to avoid commodity risk through Bitcoin, a new financial instrument.

We employed various quantitative techniques, both non-parametric (Kendall-plots) and parametric (three kinds of copulas and stationary bootstrap for the partial cross-quantilogram), to test the tail-dependence structure between Bitcoin and US oil returns. Our results show that Bitcoin has strong left-tail dependence with US oil, which means clayton copulas capture (the movement in) these variables. Furthermore, the stationary bootstrap for the partial cross-quantilogram test shows evidence that these variables share left-tail distribution at the low quantile,${\tau }=0.1$. This phenomenon comes from the flight-to-quality and inappropriately diversified portfolios. The robustness of the multiplier bootstrap-based goodness-of-fit test is employed to ensure that the Clayton copulas (left-tail distribution) capture this relationship.

Our results add to the existing literature for investors, policymakers as well as risk managers. For instance, it is very important to use Bitcoin as a hedging instrument against movements in oil commodities. In addition, when investors choose two kinds of assets in their portfolios, they should regularly examine their co-movements. Further, immediate action should be taken when one of these two returns experiences an unforeseen shock. We would also like to suggest some implications regarding the spillover between Bitcoin and the US oil markets for international investors, portfolio managers and policymakers. For instance, portfolio management and hedging strategies using Bitcoin as well as oil commodities need to take account of ‘bad’ news and information.^9^ Investors should pay careful attention to adverse information, ‘bad news’, regarding uncertain policies or a crash. More importantly, investors need to design the most appropriate strategies for hedging against a downside trend in these assets.

We suggest that policymakers also pay attention to these two markets in terms of any kind of information, because bad news might adversely influence their returns at extreme values, with a joint probability of a downside trend. When one of two markets has moved in an unusual pattern, this is a good forecasting tool for policymakers.

Our paper sheds light on the left-tail dependence between Bitcoin and US oil returns using different methodologies. There have been very few studies of this pair of investment assets. This paper does, though, have some limitations. First, we examine only the relationship between Bitcoin and US oil. Other variables would be of interest, such as the WTI index, derivatives on energy commodities, etc. Secondly, one of the new approaches for testing for a dependence structure is entropy in statistics, and research on that would be interesting. Finally, our results suggest that investigation of the influence of economic and policy shocks on structural breaks in Bitcoin and US oil returns would be interesting.

## Footnotes

1 From Reuters (https://www.reuters.com/article/us-usa-trump-oil-idUSKCN1NQ1M3).

2 The few country analyses include Hammoudeh et al. (2004) for the US, El-Sharif et al. (2005) for the UK markets, Park and Ratti (2008) for European countries, Papapetrou (2001) for the Greek market, Maghyereh and Al-Kandari (2007) for GCC countries, Cong et al. (2008) for China, and Narayan and Narayan (2010) for Vietnam, with ambiguous empirical results. Lanza et al. (2005) also investigated the relationship between stock returns and oil and gas, whereas Nwala (2007) employed further quantitative techniques.

3 The CME group also introduced this market to investors (https://www.cmegroup.com/trading/equity-index/us-index/bitcoin.html).

4 Montoro (2012) and Natal (2012) asserted that US oil shocks could be an indicator of monetary policy trade-off, which means that policymakers need to choose between inflation and stability.

5 Many previous papers use this methodology for Bitcoin returns, such as Jiang et al. (2018) and Sensoy (2019).

6 $r_{t}  = ln\left( {\frac{{P_{t} }}{{P_{{t - 1}} }}} \right)$ in which P_t_ is the index at time *t.*

7 The word ‘copula’ derives from the Latin for ‘a link, tie, bond’.

8 The results are available upon request.

9 Our suggestions are supported by the following studies: Arouri et al. (2011), Nazlioglu et al. (2013), Broadstock and Filis (2014), Ewing and Malik (2016), Kang et al. (2014), Wang et al. (2016). We employ different kinds of quantitative techniques to test the dependence structure of these variables (Bitcoin and US oil prices) and confirm the left-tail dependence structure.
